# Elevated factor V activity and antigen levels in patients with Covid‐19 are related to disease severity and 30‐day mortality

**DOI:** 10.1002/ajh.26085

**Published:** 2021-01-22

**Authors:** Fien A. von Meijenfeldt, Sebastian Havervall, Jelle Adelmeijer, Annika Lundström, Maria Magnusson, Nigel Mackman, Charlotte Thalin, Ton Lisman

**Affiliations:** ^1^ Surgical Research Laboratory, Department of Surgery, University of Groningen University Medical Center Groningen Groningen The Netherlands; ^2^ Division of Internal Medicine Department of Clinical Sciences, Karolinska Institutet, Danderyd Hospital Stockholm Sweden; ^3^ Clinical Chemistry and Blood Coagulation Research, MMK, Dept of Pediatrics, CLINTEC, Karolinska Institutet, Dept. of Hematology Karolinska University Hospital Stockholm Sweden; ^4^ UNC Blood Research Center, Division of Hematology, Department of Medicine University of North Carolina at Chapel Hill Chapel Hill North Carolina USA

To the Editor:

We were very interested to read the recent manuscript by Stefely and coworkers who reported markedly increased factor V (FV) activity levels in critically ill patients with Covid‐19.^1^ In this report, high FV activity levels were shown to be associated with thrombotic events, whereas declining levels were associated with poor outcome. A paper by Voicu and coworkers confirmed elevated FV activity levels in critically ill Covid‐19 patients.^2^ We have recently reported on the hemostatic profile of a Covid‐19 patient cohort.^3^ These patients had much milder disease in comparison to the cohort studied by Stefely. We were interested in investigating if elevated FV activity levels also occur in patients with milder disease. In addition, we wondered if the increase in FV activity reflects an increase in FV protein (antigen) level or whether these unusually elevated FV activity levels could be explained by an increase in specific activity rather than an increase in FV antigen.

Patient characteristics have been described previously.^3^ In short, we included consecutive patients with Covid‐19 in a single hospital in Sweden, and drew blood within 7 days of hospital admission. We measured FV activity using an automated coagulation analyzer (STACompact 3, Stago, Breda, the Netherlands) and FV antigen using a commercially available enzyme‐linked immunosorbent assay (Stago, Breda, The Netherlands) in platelet poor plasma of 97 patients with Covid‐19 and 28 healthy controls. Both FV activity and antigen were higher in Covid‐19 patients compared to controls, although the difference in antigen levels did not reach statistical significance (*P* = .072). Patients on medium or intensive care units had higher FV antigen levels compared to patients on general wards (Table 1). The FV activity levels were similar when patients were stratified according to their level of respiratory support, but FV antigen levels were higher in those patients with a higher level of respiratory support, although this difference did not reach statistical significance (Table 1). The FV activity and antigen levels were lower in those patients that died within 30 days of admission (Table 1). The specific FV activity (ie, the FV activity to antigen ratio) was similar between patients and controls, although patients admitted to higher levels of care had decreased specific activity (healthy controls vs high care patients *P* = .013). The correlation (performed by simple linear regression) between FV antigen and activity was less pronounced in patients (r^2^ = 0.10, *P* < .001) compared to controls (r^2^ = 0.20, *P* = .028).

**TABLE 1:** Factor V activity and antigen levels in Covid‐19 patients related to severity of disease and mortality

	Factor V activity (%)	Factor V antigen (%)	Factor V specific activity^a^
Healthy controls (n = 28)	107 [84–124]	81 [66–98]	1.26 [1.12–1.48]
Covid‐19 patients (n = 97)	125 [102–145]	93 [67–137]	1.33 [0.87–1.86]
*P*‐value	.002	.072	.868
**Covid‐19 patients stratified by location**			
General ward (n = 85)	124 [100–144]	92 [67–126]	1.39 [0.98–1.91]
High care^b^ (n = 12)	142 [124–149]	158 [106–184]	0.93 [0.66–1.16]
*P* value	.103	.007	.007
**Covid‐19 patients stratified by level of respiratory support**			
No respiratory support (n = 36)	124 [103–141]	92 [63–126]	1.37 [0.94–1.91]
Nasal cannula/mask ≤5 Liter O_2_ (n = 45)	125 [96–148]	92 [68–128]	1.39 [0.98–1.89]
Higher respiratory support^c^ (n = 17)	128 [103–150]	126 [61–183]	1.16 [0.83–1.60]
*P* value	.472	.173	.446
**Covid‐19 patients stratified by 30‐day survival**			
Survivors (n = 87)	126 [61–183]	97 [68–130]	1.35 [0.92–1.89]
Non‐survivors (n = 10)	97 [78–123]	59 [51–166]	1.32 [0.86–1.77]
*P* value	.030	.380	.752

*Note*: The results are presented as median [interquartile range]. Comparisons were made using the Mann–Whitney *U* test or Kruskal‐Wallis test, as appropriate. *P* values <.05 were considered statistically significant.

^a^ Factor V specific activity is defined as the FV activity to antigen ratio.

^b^ Three patients were admitted to the intensive care unit and nine patients were admitted to the intermediate care unit.

c Respiratory support in this group comprised >5 L O_2_ by nasal cannula/mask (n = 13), non‐invasive ventilation (n = 2), and intubation (n = 2).

Our data confirm and extend data by Stefely and coworkers. Hospitalized patients with Covid‐19 have elevated FV activity levels, with more pronounced increases in patients receiving higher levels of care. Nevertheless, our data show that even those patients admitted to general wards and patients that do not require respiratory support have elevated FV activity levels. Interestingly, we find decreased FV activity levels in the first week of admission in patients that died within 30 days of admission, which is also in line with the observation of Stefely and coworkers that declining FV activity levels appear associated with a poor prognosis. No thrombotic events occurred in our cohort during a 30‐day follow up, and we were therefore unable to confirm the finding by Stefely of elevated FV activity levels as a risk factor for Covid‐19‐associated venous thrombosis.

Elevated levels of FV activity were in part related to increased FV antigen, although the correlation between FV activity and antigen was modest in both patients and controls. Besides fibrinogen, FV is the only liver‐derived coagulation factor that shows elevated levels in Covid‐19 patients, and the reason for this selective FV increase remains unclear. Part of the FV in patients with Covid‐19 may be released from platelets, which are known to be activated in Covid‐19.^4^ Megakaryocytes endocytose factor V from plasma, and proteolytic modification of FV results in a factor V molecule with procoagulant properties that are distinct from and more thrombogenic than plasma FV.^5^ The more pronounced increase in FV activity compared to FV antigen in patients, suggests FV in patients with Covid‐19 to be hyperactive, but the specific activity of FV was not different between patients and controls. Interestingly, the specific activity of FV was slightly (but not significantly) higher in patients on general wards compared to controls, whereas in patients receiving higher levels of care, the specific activity of FV was decreased compared to controls. We thus hypothesize that part of the FV in patients with Covid‐19 is platelet‐derived, but is in part inactivated in sicker patients.

In conclusion, FV displays unusual behavior in patients with Covid‐19 and we concur with Stefely and coworkers that studies on the value of FV as thrombosis biomarker and prognostic indicator are warranted.

## CONFLICT OF INTEREST

The authors have no conflicts of interest to disclose.

## FUNDING INFORMATION

C.T. received funding for this study from Region Stockholm, and Knut & Alice Wallenberg foundation.

## ETHICS APPROVAL AND PATIENT CONSENT STATEMENT

The study complied with the declaration of Helsinki, and informed consent was obtained from all healthy individuals and patients, or in the case of incapacity, their next‐of‐kin. The protocol was approved by the Stockholm Ethical. Review Board (COMMUNITY study dnr 2020–01653).

## Data Availability

The data that support the findings of this study are available from the corresponding author, upon reasonable request.
